# Hospital and Institutional News

**Published:** 1918-04-20

**Authors:** 


### HOSPITAL AND INSTITUTIONAL NEWS.

### NOT KILLED BUT A PRISONER.

Lieutenant-General Sir Arthur Sloggett, D.G., A.M.S., whose courteous hospitality to all whose duty and privilege it has been to visit the Armies in France is greatly appreciated, recently received the intelligence that his only son, Lieutenant-Colonel A. J. H. Sloggett, D.S.O., Rifle Brigade, had been presumed killed in action. The latest dispatches contain, we rejoice to note, the welcome announcement that Colonel Sloggett is alive, though unfortunately a prisoner at Carlsruhe. Warm sympathy and congratulations will be extended to Sir Arthur and Lady Sloggett in these happier circumstances, and the general wish will be that their only son may speedily be freed from the irksome lot of a prisoner. Colonel Sloggett was in command of a battalion of the Rifle Brigade, which on March 21 was known to have been carrying on a desperate fight in holding up a German division, and he was subsequently reported missing.

### THE WAR OFFICE AND BRISTOL ROYAL INFIRMARY.

With a loss on the year's working of £14,000, so far as the ordinary income is concerned, the Bristol Royal Infirmary is in an awkward financial condition. This is partly due to the realisation a few years ago of most of the capital in order to build the new surgical block, which is now devoted to the wounded. The idea of the late President, Sir George White, was to make up the income from annual subscriptions. In this he was partly, but far from wholly, successful. A big legacy a few years ago, if we remember, seemed to retrieve the situation, but the present position shows that more remains to be done. It is hoped to increase the workmen's contributions, and, further, since the new block is used entirely for the wounded, to complete the recent negotiations with the War Office for an increased grant. This grant, it is understood, will be retrospective. It is even suggested that the infirmary should be recouped, not only for the cost of the men's maintenance, but also for the loss of revenue which was occasioned by the realisation of investments, on the ground that this loss was incurred to build the great wing which the men now wholly occupy. The War Office is necessarily careful of making precedents, but there is a chance of an important concession. Much, however, remains to be done in this and other directions.

### A NEW HOSPITAL FOR LONDON.

Woolwich confesses itself to be the only town of its size and importance which does not possess or maintain a general hospital. Advantage is being taken of the prosperity which has come to it through the war to start a building fund of £50,000 for a hospital of 100 beds. A successful fair has been held to give the fund a send-off with £3,000 or so, and on the first day Lord Burnham, a trustee of King Edward's Hospital Fund for London, said that he would do all he could to make the grant fully equal to Woolwich's own effort. The chairman of the building fund is Mr. George Whale, who had the good sense to remark that the only worthy war memorial of the armies would be an Homeric or Miltonic epic, but, since this could not be guaranteed, the occasion might be improved, in Woolwich at all events, by the building of a hospital to commemorate those fallen in the war. Outer London had no adequate hospital accommodation, and its needs were greater than ever at this time. There is an endurance in the Arsenal as well as in the trenches, and if disputes mar the work of the former, as Mr. Whale pointed out, the factory has not the stimulus of actual conflict. The proposal for a hospital in Woolwich can hardly fail to meet the success which it deserves.

### CHICHESTER AND SECTIONAL SUBSCRIPTIONS.

Notwithstanding the fact that the Royal West Sussex Hospital, Chichester, has received £462 from the appeal issued to liquidate the deficit of £894, about half this sum still remains unpaid, and legacies will probably be employed to meet it. The report has shrunk to a single folio sheet of four pages, and those who wish to study the hospital's statistics and list of subscribers and donors must pay a visit to the secretary's office. The continued absence in France of Captain A. H. Bostock, R.A.M.C., has added to the work of the staff, though Mr. G. Lansdown, the honorary overseer, and Dr. B. E. Wright, the house surgeon, have done their best to help "carry on." Approximately one-third of the 1,066 in-patients have been sick and wounded soldiers, payments for whom have amounted to £1,509 from the War Office. Out of an ordinary income of just over £7,000 nearly £2,000 was derived from annual subscriptions, which, with the War Office contribution, have provided half of the ordinary receipts. This is not a bad record, but the aim should be to raise the total from annual subscriptions to £3,500. If this could be done the average present deficit would be more than met, and legacies would be saved for capital. Is it not possible to start a definite organisation to form a farmers' and a Chichester Workers' Fund to help the annual subscriptions? What is called a Workers' Fund has proved a success at Cambridge, where the conditions are somewhat similar to those of Chichester. Here is a chance for Mr. Tom B. Harrison, the secretary, at all events.

### AN OPPORTUNITY FOR ABERDEEN.

The directors of the Royal Aberdeen Children's Hospital have decided to reopen the building fund, which was suspended soon after the war because of the appeals which then began to flood the country. The timid policy which led to the appeal being suspended was understandable, but has proved short-sighted, since events have proved that, war or no war, there is no time like the present for raising money. The wealth of the country has never been more apparent than in these dark days, when bankers still lament the huge balances lying idle on current account and the reluctance of many persons to transfer them to the Treasury through the purchase of War Loan. We therefore hope that this appeal will be resumed with vigour, so that the bigger and better premises which the Children's Hospital needs may be provided for before the after-war period, when, for all we know, money may be much harder to find than it is at present. If it is decided to adopt the device of a war memorial, well and good, but at all events let Sir Thomas Burnett, Bart., beware of letting slip the present and immediate opportunity.

### THE CASE FOR A HIGHER GOVERNMENT GRANT.

An appeal for a more liberal allowance from the Government for the treatment of soldier patients was made by Mr. Thomas Woodsend, the president, at the annual meeting of the Liverpool Royal Southern Hospital, last month. After pointing out that the seventy beds reserved for sick and wounded soldiers had been regularly occupied, he said the cost of each in-patient per week during 1917 worked out at 41s. 2d. as compared with 32s. 8¾d. the previous year, and although the Government had recently intimated its willingness to increase its grant to 4s. 9d. a day, or 33s. 3d. a week (instead of 28s.), that was not enough. The Government allowance, he contended, should be 6s. a day, 42s. a week, for he feared that the continuing rise in prices would have a further adverse effect on working costs during the current year. Mr. Woodsend said that they felt that the Government allowance should at least cover the cost and that subscriptions and gifts should be devoted to civilian cases, which had as great and as sacred a claim on them. This puts the case of the voluntary hospitals with admirable simplicity and directness, and is a model statement of their claim.

### A HOSPITAL ESTATE.

It is probably safe to say that no hospital in the world has more extensive grounds than the Princess Louise Hospital for Limbless Sailors and Soldiers at Erskine, in the West of Scotland. Thanks to a recent gift by Mr. John Reid, the grounds now extend to 500 acres. The added area includes a wood of fifty acres and fortyfive acres of agricultural land, which Mr. Reid has purchased and presented to the hospital trustees in order to prevent it falling into other hands at any time during the future. It should be possible, on this forty-five acres, to grow all the vegetables needed for the institution and a considerable portion of the cereal foods also. It was Mr. John Reid who in 1916 provided the funds which enabled Erskine House to be secured for its present purpose.

### THREE GIFTS TO THE RAMSGATE GENERAL HOSPITAL.

ONE of the two private wards on the women's side of the above hospital is to be called the Dame Janet Stancomb-Hills Ward in recognition of the honour conferred on Miss Stancomb-Hills by the King. Apart from her annual subscription and smaller donations, Miss Stancomb-Hills has given the hospital £2,000 to name two beds in the general wards, one on the women's side in memory of her aunt, known as the "Lady Hills of Blaydon Bed," 1916, and one in memory of her uncle, on the men's side, which is called the "Lord Winterstoke Bed," 1915. Mrs. Richardson, of Somerset, sister of Dame Janet Stancomb-Hills, has paid for the new lift to the raid shelter (the patients are now moved on their beds), saying when she sent her cheque that she thought the people living in the West in comparative safety should help those on the East Coast, who were living more or less in danger. From the Officers' Mess at the Menstone Aerodrome the committee has received a cheque for £25. The hospital has taken the acute cases and serious accidents from this Aerodrome from when it was first started. The staff much appreciates this gift, as Navy officers and men have been told when in hospital that they were paid for by the Government.

### PROPOSED STANLEY MAUDE CONVALESCENT HOME.

WITH the approval of the Secretary of State for War and of the Minister of Pensions, it is proposed, on the initiative of a few of his relations and friends, to establish a convalescent home for maimed or crippled soldiers as a national memorial to the late Lieutenant-General Sir Frederick Stanley Maude. Those who served under him in Mesopotamia would have a prior claim to admission, and the intention is to provide not merely an institution, but a real home for the men, where they would find comfort as well as care, and country air as well as training for a livelihood. Donations may be sent to Messrs. Cox and Co., 16 Charing Cross, S.W. 1, and it is hoped that the scheme, by benefiting the crippled or maimed in the later stages of their recovery, will prove a worthy national memorial to Sir Stanley Maude's services and perpetuate his name.

### PAY WARDS IN COTTAGE HOSPITALS.

THE satisfactory financial position of Beckenham Cottage Hospital, which ended its year with a balance in hand of £375, is not only due to special donations. Fees from paying patients have amounted to £230, which shows that the private ward has been almost continuously in use. So long as these wards do not encroach on the beds required for the general public, and the fees are kept low, they fulfil a very useful purpose and add to a cottage hospital's repute. It would be a mistake to regard them as a source of income, except in so far as all payments received can, of course, be set against expenditure.

### THE RED CROSS SALE AND QUEEN MARY'S EXHIBITION.

THE fourth Red Cross Sale at Christie's, which began on April 8, will last till April 25, and again a surprising number of interesting and important objects have been collected and given. The catalogue, whose price of half-a-crown also benefits the Red Cross Fund, is again, therefore, interesting reading, and a visit to the sale-rooms is well worth paying even by those who are unable to bid. Another important exhibition to which the Queen has given her name is that of the work carried out by wounded and discharged sailors and soldiers which is to be held, on a date, not yet fixed, at the Central Hall, Westminster. The Pensions Minister, Mr. Hodge, attaches great importance to the exhibition as a means of encouraging disabled men to learn new occupations, and thereby to add to their pensions. The first exhibition of the kind was held last June at Messrs. Sotheby's new rooms, in New Bond Street. The head of the firm, Sir Montague Barlow, is the chairman of the exhibition committee, and the firm is again lending its services.

### GIFTS OF PLATE FROM GRATEFUL PATIENTS.

ON the temporary closing of the Priestley Green Auxiliary Hospital, Lightcliffe, Yorkshire (see THE HOSPITAL, April 6, p. 5), the Sister and the V.A.D.s have each received a present of a piece of plate from recent patients. The gifts, which came as a pleasant surprise, were accompanied by messages of gratitude, and have caused much pleasure. This unofficial testimonial to the patients' appreciation is more valuable than a formal presentation.

### WORDS AND THINGS: A CASE IN POINT.

SOME months ago several Boards of Guardians decided to alter the name of "workhouse," substituting "institution" instead. Now the London County Council Asylums Committee has been induced to abolish the title of "lunatic asylum" in respect of the Ewell Colony, which will continue to be known by its present name, and to designate all its other institutions "mental hospitals." Several London Boards of Guardians had requested that the Committee should make this alteration, which no doubt will diminish in time such disfavour as existed in reference to these places. Certainly the new title of "mental hospital" will sound less obnoxious to many sensitive persons who have the misfortune to have either relatives or friends in them. It is easy, as we lately observed elsewhere, to make a case in principle against these rechristenings, and to point out that if the function of a thing remained unchanged, each new name in time will suffer from any odium with which the function may be charged in the public mind. The word "asylum" sounded an immense improvement on "mad-house," but "asylum" is now to be changed. Modern immodesty has left no name for a place where a man may wash his hands, without suggesting that he really seeks a closet. There is no arguing with these absurdities. Each case has to be decided on its merits, and the alteration of the word "asylum" has perhaps a better case for it than most. Besides, case or no case, it was bound to come anyway.

### EXPOSURE DURING AIR RAIDS.

The necessity for people to remain in their own homes when warning of an air raid is received is again borne out, this time by Dr. G. Millson, medical officer of health for the borough of Southwark. Some alarming statements have been made about the general health of the borough during the first two months of the year, and consequently the Public Health Committee took the matter into full consideration. Dr. Millson presented a special report to the committee, pointing out that of eighty-eight deaths of children under a year old, and 114 between one and two years old, forty-two were attributable to the children being taken out at night to air-raid shelters. Another notable feature of the report was the large number of deaths of persons over forty-five years of age. They numbered 311 out of a total of 633, and, strange to say, the deaths of males considerably out-numbered those of females. Dr. Millson states that the stress of war and exposure during air raids were responsible for the increased deaths of infants and elderly people. Upon the whole he was of opinion that the death rate for Southwark in the period under review—20.6 per 1,000—was not excessive for the time of the year. The births, however, were less than the deaths in this period, for they only amounted to 569, giving a birth rate of 18.5 per 1,000. Dr. Millson, however, points out that these short periods of statistics were not satisfactory, though they might be necessary, and that no period under a complete year could enable a correct comparison to be made of the health of the district.

### AN AIR-RAID COT.

The Poplar Hospital for Accidents has received the sum of £500 to endow a cot in memory of the children who were killed during the air raid of June 13 last. There is something peculiarly fitting in such a memorial. The air-raid cot, as it might properly be called, will remain as a dignified memorial of events not easy to imagine when once the period of their occurrence is over. But, apart from this, a cot so dedicated will speak, as few such cots can do, of the part played by the hospital itself during the Silvertown and other explosions—a London hospital often in the firing line! The name of the hospital has proved to be more aptly chosen than its founders could guess. Air raids are "accidents" in the East End, throughout which the hospital has maintained its high record.

### ST. JOHN AMBULANCE BRIGADE AT BIRMINGHAM.

When, if ever, the whole history of the work of the St. John Ambulance Association and the St. John Ambulance Brigade during the war comes to be written with any detail it will be found that the activities of these bodies in Birmingham have not lagged behind those in other industrial centres. At the annual meeting in the Council Chamber, Birmingham, Sir John Holder, president of the Association, in moving the adoption of the report covering two years from 1914 to 1916, described how an organisation, designed primarily for civil work, had played a great part in the war. In Greater Birmingham there are now twenty-seven Voluntary Aid Detachments and seven St. John hospitals, in which 800 beds had been established as part of the Birmingham directorate. In these hospitals the whole of the nursing and domestic work is being done by V.A.D. members. In respect of the ambulance and transport work, it was stated in the report that 168,000 had received much-needed refreshment at Snow Hill Station, and in two years nearly 300 ambulance trains, carrying over 30,000 wounded soldiers, were attended by the transport sections. It was now possible to unload a hospital train, containing mostly stretcher cases, in under fifty minutes.

### CONSUMPTIVES AND THE FOOD RATIONS.

A curious point is noted in Dr. Robertson's report on the progress of the patients at the Royal National Hospital for Consumption, Ventnor. In the years from 1916-17 the patients increased in weight more rapidly and in a greater degree than in the pre-war years 1912-13. A pound of weight was gained in twelve days, whereas fourteen days were required for this gain before the war. It is suggested, in the absence of direct evidence, that the explanation may be due to a lower average standard of nutrition on admission. It is true that the hospital has had to reduce its consumption of food, but, even so, the diet supplied to the patients was, in Dr. Robertson's words, much in excess of that of the outside population. One could understand that weight might be put on more quickly in hospital when patients were suffering from unusual malnutrition on admission, but it is strange that they should have put on more weight on a reduced hospital diet. What is the explanation?

### PATIENTS AND SANATORIUMS.

At a recent meeting of the Staffordshire, Wolverhampton, and Dudley Joint Tuberculosis Committee it was decided to try to lease two large residences and adapt them as sanatoriums, in order to replace the institution formerly used for that purpose, with regard to which there had been complaints from the patients. Whether such complaints were justified or not, it would certainly appear that the inmates did not remain long in residence, for whereas the number on the books was about fifty, those leaving every month to be replaced by newcomers averaged about thirty. This resolution represents in short an endeavour to retain consumptives in sanatoriums for a proper period by increasing the amenities of such places. The cost of adapting one private residence was given as £3,250, and, as the Clerk to the Committee pointed out, this was a considerable expenditure on premises taken at an annual rental. Decisions of this kind bear out a contention often advanced in these columns, that the status of the sanatorium, and particularly of its resident medical head, badly needs raising. A few large central institutions are infinitely more efficient and satisfactory and economical than a host of little separate endeavours to accommodate a few victims of tuberculosis. The Local Government Board's approval is of course, in this time of makeshift, not likely to be withheld from these projects, but with the advent, post-war, of a Ministry of Health, one may hope to see a Tuberculosis Department thereof with a centralised scheme of administration and full powers to apply it. Parochialism may do very well for things humanistic, for our friends the lawyers and the educationists, but it will not do for medicine, because medicine deals with Nature, and Nature will not wait or stay her hand out of pity for human stupidity.

### MUNICIPAL WORK TO ENSURE PURE MILK.

BIRMINGHAM is attempting to eradicate tuberculosis from dairy herds of the city, and thereby to ensure a pure milk supply for the people. At the end of last year there were 181 dairy farms, 357 cowsheds, and 2,176 dairy cows in the city under the supervision of the Corporation's veterinary department. Twelve cows were found to be suffering from tuberculosis. Eleven were forthwith slaughtered; the twelfth was kept alive in isolation, because it happens to be a valuable pedigree animal in calf. It is hoped that the calf will be free from the disease. Twenty cows had catarrhal mastitis or catarrhal inflammation of the udder, and five were emaciated. The milk of all these cows was prohibited from sale, either temporarily or permanently. At the end of the year twenty-five herds, comprising 749 cows, were being dealt with under the scheme. Of these twenty-two herds, numbering 692 cows, were free, and the other three herds, numbering fifty-seven cows, were being freed. The Government has differentiated between the price to be paid for milk from cows certified as being tubercle-free and that to be paid for milk of cows not so certified. All Birmingham dairymen whose herds have been certified as tubercle-free are now obtaining the higher price for milk.

### THE STATUS OF DENTAL SURGERY.

SINCE the war dental surgery has begun to receive its due, and this reminds us of the honour which should be given to the believers in this branch of medical science in the not remote days when a dentist was almost a term of contempt. Even now, after thirty-eight years of work, we learn that the public is still mostly ignorant of the benefits provided by the Devon and Exeter Dental Hospital, in spite of the success of the dental scheme, which now has solid experience behind it. The nature of this hospital's work can be guessed from the fact that its income was £200 last year, and that the number of cases treated was 930. How far is the enrolment of men in the Army, which is supposed to look after the soldiers' teeth properly, responsible for the falling off in the number of cases treated? At all events, 930 patients is only two-thirds of the number recorded for 1916.

### A HEALTHY WAR HOSPITAL.

THE first year of work is practically complete at the Redhill War Hospital, which the Reigate Poor-Law Infirmary became when the Army Council requisitioned it in January 1917. Though the building was new, having only been occupied in 1916, £657 had to be spent in new buildings and alterations, especially in the erection of a temporary kitchen. The existing kitchen, it is instructive to note, was found wholly inadequate! However, in February 1917 the Redhill Women's V.A.D., No. 108 Surrey, was asked to staff, administer, and finance the institution. In March a successful appeal was issued. Early in May the nursing staff was installed, and on July 3 the first convoy of patients arrived. Colonel Rawson gave his Parliamentary salary, Miss Woolley presented a pony and an invalid chair, Hull stabled it, and gifts in kind of game, fruit, vegetables, and dairy produce were generously provided. The first year ends with a substantial balance in hand, part of which is to be used as a sinking fund for reconverting the hospital to the uses of the Guardians at the end of the war. The patients numbered 228, and stayed on an average about six weeks. So this war hospital of eighty beds has made a healthy start, and deserves the continued support of its friends and sympathisers.

### MODERN BOOKS FOR SOLDIERS.

IN response to a request from the wounded soldiers in the Great Northern Central Hospital for guidance in reading modern English literature, a lecture on "A Few Modern Books" was lately given in the Military Annexe, Manor Gardens, by Mr. A. Rowan, Normal Master, L.C.C. Shoreditch Technical Institute. The authors chosen for remarks and reading of selections were Kipling, Masefield, and Watson. Reference was made to Kipling's "Barrack Room Ballads," etc., and some parodies of Kipling's style, not altogether complimentary to the original, were read, after which the lecturer proved from selections from such books as "Puck of Pook's Hill," "Rewards and Fairies," and Kipling and Fletcher's "History" that the strictures in the parodies did not apply to later work, however much they might have applied twenty years ago. Some other amusing parodies of Kipling, Tennyson, and Burns were read to direct attention to such writers as Russell in "Collections and Recollections," etc. The lecturer then directed attention to such poems as Masefield's "Everlasting Mercy," "The Widow in the Bye Street," "The Dauber," and "Daffodil Fields," and such portions as might lead to a desire to know more of these were read. The men were keenly interested and delighted, and asked for the loan of all of the books mentioned. These have been kindly lent by Mr. W. J. Harris, of the Islington Central Library, and are much appreciated. There was not time to deal with William Watson, but a lecture was promised for a subsequent date.

### DIPHTHERIA CASES IN DEVONSHIRE.

Certain cases of diphtheria have occurred at Hawkmoor Sanatorium, Devonshire, of which Dr. Mapleton, medical officer of health, is sending a full report to the Local Government Board. At the same time a five-year-old child, suffering from diphtheria, from which it has since died at Elsford, Bovey Tracey, was refused admission to the Isolation Hospital on the since disproved ground that its home was not in the hospital area. The Newton Abbot Rural Council, dismayed at this, have passed a resolution asking the isolation hospital authorities to modify their rules so that urgent cases might be admitted, and the question of payment be afterwards arranged. It is a pity that the matron had not the courage to act on her own initiative and admit the child.

### THE SALARIES OF MASSEUSES.

In a letter to the Bradford Corporation Lt.-Col. Wrangham, O.C., War Hospital, Bradford, states that, as the members of the Almeric Paget Military Massage Corps were now paid £2 10s. a week and were allowed a week's holiday every quarter with pay, the masseuses employed at the hospital would like an increase of their present maximum rate of pay of £2 a week to £2 10s. and similar holiday arrangements. Acting on behalf of the Ministry of Pensions, the War Pensions Committee of the Corporation has decided to concede the request.

### TWO APRIL "GAZETTES."

Among the news contained in the April number of the *Gazette* of the 3rd London General Hospital, Wandsworth, is that of the success of the War Savings Association of the unit. Nearly £4,000 was raised in the first five days, and an endeavour is being made to show a good monthly record. The idea was started by Staff-Sergeant Mountain and Corporal Green. Corporal Edwin Martin contributes a full-page sketch entitled "The Clock Tower." There is a skit on Mr. Nevinson's popularity as an artist, and such skits are the proof of his success. There are, too, several amusing pen-and-ink drawings, and some hospital photographs, but the letterpress on this occasion does not call for detailed comment. We have received a copy of the current issue of *The Pavilion Blues*, the magazine of the Pavilion Hospital, Brighton. Edited by Colonel Coats, C.B., with Sergeant G. J. Penfold as sub-editor, it is now well into its second volume. On the whole the magazine confines itself to chaff, though, in view of the material which has been found at, for instance, St. Dunstan's, where a most interesting magazine exists, we should have thought that the Pavilion Hospital, which treats, of course, another kind of special disability, would have found sufficient points of interest in the work of the institution to provide some articles to season the jokes. It is true that Colonel Maurice, C.M.G., the Commandant, writes on Birds in this number, a subject on which he is a modern Gilbert White. But, apart from his brief notes and a summary of his recent lecture, the magazine seeks to raise a smile and nothing more.

### THE BRITISH PHARMACOPŒIA IN WAR-TIME.

For the second time since the beginning of the war certain important changes have been made in formulas of the British Pharmacopœia. On both occasions these changes became expedient because of the necessity of reserving exclusively for use as foodstuffs or essential war purposes substances which were also used in medicine. The first alteration affected preparations containing glycerin and sugar, and the second Order, which has just been made by the General Medical Council, has been necessitated by the shortage of olive oil, lard, suet, and refined castor oil. This new Order, which takes effect at once, provides that Pharmacopœial liniments prepared with olive oil may now be made either with arachis oil or sesame oil, and that preparations which have hitherto been made with lard or suet may now be made with lanoline, either singly or mixed with hard paraffin, liquid paraffin, soft paraffin, paraffin ointment, white beeswax, yellow beeswax, or any combination of these as may be necessary to impart a proper colour and consistence to the product; the official proportion of the active ingredient must in all cases be maintained. As regards castor oil, the Order provides that a second quality of oil may be employed in making preparations instead of the finest quality—which latter is required for very important war purposes. Hospital pharmacists will have no difficulty in meeting the emergency.

### THIS WEEK'S DRUG MARKET.

Almost all drugs have an upward price tendency at the moment, the exceptions being very few, and even then the exceptional cases are due to causes which may only be temporary. As was anticipated, English refiners of camphor have again raised their quotations, and in view of the fact that they cannot supply except in limited quantities, it seems probable that there will be a further advance before long. Cocaine is scarcer, and the price is again higher. There is a good demand for iodine preparations at full rates. The large demand for hypophosphites cannot be satisfied owing to scarcity. Hexamine, acetanilide, and formaldehyde continue scarce. Extremely high prices have been paid for calumba root and Siam gum benzoin. A large business has been done in imported honey at prices slightly below those which had been paid until quite recently; prices, however, are still extraordinarily high, and likely to remain so. Acetylsalicylic acid, phenacetin, and paraldehyde have a slightly easier price tendency. Remarkable prices are asked for olive oil; arachis oil, which the General Medical Council now allows to be used in Pharmacopœial preparations instead of olive oil, is very difficult to obtain.

